# Chronic Total Occlusions: New Pathways to Success

**DOI:** 10.2174/1573403X1002140506123519

**Published:** 2014-05

**Authors:** Emmanouil S. Brikalis

Every day seems to bring a new development in the treatment of chronic total occlusions (CTOs). The interest in this field is to be welcomed, given the rapidly increasing procedural success rates, in spite of attempting CTO percutaneous coronary intervention (PCI) in increasingly complex patients and lesions [1].

Yet the barriers to treatment for this group remain formidable and include sometimes long procedure duration and cost [2], perception of higher complication rates [3], and lack of clinical evidence to support CTO PCI, include procedures, that on average, take longer, cost more and have higher complication rates. There is also a perception that there is a relative lack of clinical evidence to support CTO PCI.

Given that angiographic markers of complexity predict longer procedures with higher incurred costs, it may be that the higher complexity (and thus need for strategical diversity) causes a reflection on the prognostic benefit of the procedure that does not seem to defer non-CTO PCI [4]. Yet it still seems likely that the biggest barrier to more widespread uptake is the reproducibility and applicability of newer methods for teaching CTO PCI.

This series of commissioned articles aims to review the evidence base of CTO PCI, define the evolution of techniques with particular reference to the pathophysiology of the disease and describe how techniques have evolved to meet the challenges referred to above. It defines the role for pre-procedure planning in treating this complex lesion subset, describing the role for adjunctive imaging both in planning and in ensuring optimal procedural results. The chronicity of these lesions carry specific challenges and newer techniques for treating coronary calcium and ensuring both equipment delivery and optimum stent deployment are also covered.

It is clear that success in the CTO PCI environment can no longer be claimed by individual, relatively small series, but should be viewed more in terms of growth in the overall percentage of CTO PCI patients. The challenge is to ensure that anatomical factors do not deter physicians from percutaneous revascularization where clinical indications support it.
